# Data on the occurrence of corticolous myxomycetes from Denali National Park, Alaska

**DOI:** 10.1016/j.dib.2016.03.048

**Published:** 2016-03-18

**Authors:** M. Schnittler, N.H.A. Dagamac, M. Sauke, M. Wilmking, A. Buras, S. Ahlgrimm, P. Eusemann

**Affiliations:** a Institute of Botany and Landscape Ecology, Ernst Moritz Arndt University Greifswald, Soldmannstr. 15, D-17487 Greifswald, Germany; b Institute for Stochastics, TU Bergakademie Freiberg, D-09596 Freiberg, Germany; c Johann Heinrich von Thünen-Institute of Forest Genetics, Eberswalder Chaussee 3a, D-15377 Waldsieversdorf, Germany

**Keywords:** Amoebozoa, Myxomycetes

## Abstract

This data set contains data about corticolous (bark-inhabiting) myxomycetes from a 100×100 m^2^ plot including ca. 380 trees of *Picea glauca* (white spruce), of which 260 were large enough that bark could been sampled to prepare moist chamber cultures. At the end of the data set records of myxomycetes from 66 moist chambers prepared with bark of deciduous trees and shrubs, and outermost twiglets of *P. glauca* are included. These were sampled around the plot for purposes of comparison. A second data set shows measured tree parameters for the 380 trees examined in the plot. Data were used for a statistical analysis to search for environmental factors decisive for the occurrence of corticolous myxomycetes (Schnittler et al., 2016) [1].

**Specifications Table**

**Table:** 

Subject area	Biology, Mycology, Protistology
More specific subject area	Records of protists: Plasmodial slime molds, Amoebozoa
Type of data	Table
How data was acquired	Applying the moist chamber culture technique to bark sampled from trees and shrubs
Data format	Record data, specimens determined
Experimental factors	Features of the trees where bark was sampled were recorded (age, dbh, vitality etc.).
Experimental features	Myxomycetes occurring in the cultures were recorded by examining them with a dissecting microscope at seven occasions.
Data source location	Location of the plot: USA, Alaska, Denali National Park, Rock Creek watershed, ca 1.2 km north of the park access road (149 °00′36″ W, 63 °43′29″ N).
Data accessibility	Data is within this article

**Value of the data**

• The data contribute to further knowledge of myxomycetes from Alaska, especially regarding bark-inhabiting (corticolous) myxomycetes.
• The data can be used to enlarge the checklist of myxomycetes for the region.
• So far, data have been analyzed in an attempt to determine the factors decisive for the occurrence of corticolous myxomycetes [1].

## 1 Data

The data file in the format Excel 2010 contains two spreadsheets; the first showing the records of myxomycetes, the second the parameters measured or estimated for the trees of *Picea glauca* in the 1 ha plot. All columns are explained by comments in the heading field. Specimens were determined according to standard literature, nomenclature follows [2].

Voucher specimens were deposited in the Botanical State Collection Munich (M), with duplicates of selected specimens in the herbarium of the University of Arkansas (UARK).

## 2 Experimental design, materials and methods

All 380 trees of *Picea glauca* in the selected plot of 100×100 m^2^ were mapped with a differential GPS (Trimble R3) in July 2012, allowing a precision of <=30 cm (floating mode and post processing).

From 260 of the 380 *Picea glauca* trees in the plot about 20–30 bark pieces around each trunk (to minimize possible differences related to different exposition of N-or S-facing sides of the trunk) were sampled between 1.2 and 1.5 m height; This height was chosen to exclude soil-inhabiting myxomycetes, where plasmodia often migrate to elevated points to fruit. Sampling included nearly all larger trees (dbh>4 cm); the majority of the 120 trees not sampled was below 0.5 cm dbh or were saplings not reaching sampling height. The outermost, dead bark and bases of small dead branches were carefully removed without injuring the living part of the tree. Bark samples were air dried in paper bags and transported back in the laboratory.

In the frame of a larger study we investigated as well tree parameters, these were coded as eight environmental variables. Vitality of the trees (vit), was assessed using a five-divided scale: 1=all branches green, ending with freshly grown shoots; 2=5–15% dead branches, crown intact, fresh growth in about 70% of all outer branches; 3=15–50% dead branches, crown damaged but alive with at least half of all outer branches in fresh growth; 4=>50% dead branches, crown damaged or broken off,<20% fresh growth, tree dying; 5=tree dead. The pH of the bark surface (pH) was measured in the prepared moist chamber cultures for all 260 trees where bark was cultured. Diameter at breast height (dbh) was measured for each tree. Tree age (age), was determined using tree cores and ring counts of all trees allowing coring, altogether 175 trees. Tree height (height) was measured directly or determined with a clinometer. The area of the crown (crown) was estimated from two perpendicular measurements of crown diameter. An index of light competition (comp) was computed from crown radius (mean of two measurements perpendicular to each other), tree height and geographical position. The overlap of neighboring tree crowns was calculated as the overlap of two circles. Overlap was then inversely related to the height of the two trees in question, with the higher value assigned to the smaller tree. As such, this index estimates the degree to which one tree is shaded out by others – which should lead to a cooler and moister microclimate for this tree in comparison to others. Per cent shrub cover (shrub) was estimated in a 3 m radius around each tree.

The collected bark samples for each tree were cut into pieces of 1×1 cm and placed in a 9 cm Petri dish lined with three layers of absorbent paper towel in a way that most of the surface (ca. 60 cm^2^) was covered. Samples were completely soaked in distilled water overnight. After 24 h, excess water was removed, and the pH of three pieces was measured with an Orion 610 solid state probe. Moist chambers were incubated at room temperature under diffused light for in total 77 days and observed regularly (days 6, 11, 22, 33, 52, 64, and 77) for the presence of plasmodia and/or fruiting bodies. All fruiting bodies of myxomycetes that developed in the moist chamber cultures were recorded, determined and sometimes collected. Furthermore, the number of fruiting bodies per culture was recorded to obtain abundance data.
