# Supplementary material for: Data on the occurrence of corticolous myxomycetes from Denali National Park, Alaska
Source: Data Brief. 2016 Mar 18;7:1196–8. doi: 10.1016/j.dib.2016.03.048 (PMC4927954; doi:10.1016/j.dib.2016.03.048)
Supplement: Supplementary file 2 — Supplementary material [file mmc2.docx]

I hereby declare that these data were collected with permission of the US National Park Service and a report was filed to the respective authorities.

I furthermore declare that there is no conflict of interest with any third persons.

Martin Schnittler, 2016-03-01
